# Reassessing metrics: The limitations of brain function as indicators of brain health

**DOI:** 10.12669/pjms.41.8.12154

**Published:** 2025-08

**Authors:** Umema Zafar, Syed Hamid Habib, Muhammad Omar Malik

**Affiliations:** 1 Umema Zafar, MBBS, MPhil, PhD. Associate Professor, Physiology Department, HBS Medical and Dental College, Islamabad, Pakistan; 2 Syed Hamid Habib, MBBS, MPhil, PhD. Associate Professor, Physiology Department, Institute of Basic Medical Sciences, Khyber Medical University, Peshawar, Pakistan; 3 Muhammad Omar Malik, MBBS, MPhil, PhD. Associate Professor, Physiology Department, Institute of Basic Medical Sciences, Khyber Medical University, Peshawar, Pakistan

**Keywords:** Brain health, Neuro-biomarkers, Cognition Disorders, Neurofilament light, Neuron Specific Enolase, Phosphorylated Tau proteins, Regression Analysis

## Abstract

**Objective:**

This study aimed to evaluate brain health using cognition, vision, hearing, emotional well-being, aging, and motor function as predictors, with blood neurofilament light (NfL), neuron-specific enolase (NSE), and phosphorylated tau (pTau) as response variables for detecting chronic brain injury.

**Methodology:**

This is a predictive cross-sectional modelling study carried out in various settings across Peshawar, Pakistan in which a total of 75 healthy adults were included from May 2022 to February 2023. Various tests were performed including visual fields, audiometry, dynamometry, Mini-Mental State Examination (MMSE), and Trait Emotional Intelligence Questionnaire (TEIQue-SF). Serum levels of NfL, pTau, and NSE were measured via ELISA and multiple linear regression models were developed. These biomarkers were selected based on a preliminary scoping review.

**Results:**

Statistically significant discrepancies were present between predicted and actual biomarker values (NfL: actual 0.026±0.011 ng/ml vs. predicted 0.001±0.000 ng/ml, p<0.001; NSE: actual 10.46±1.52 ng/ml vs. predicted 4.127±1.8 ng/ml, p<0.001; p Tau: actual 4.48±3.23 ng/ml vs. predicted 1.027±0.85 ng/ml, p<0.001), indicating poor prediction model performance. The k-fold cross-validation confirmed this, with over 2% deviation in all subsets.

**Conclusion:**

Brain health prediction models based solely on neuro-biomarkers and brain function parameters may not generalize well to healthy populations, indicating the need for additional factors influencing brain health.

## INTRODUCTION

The brain is made up of billions of interconnected neurons that enable us to process thoughts, experience complex emotions, and perform motor functions. A healthy brain is essential for overall well-being, quality of life, and longevity. However, despite its significance, the concept of “brain health” remains vague, with no universally accepted definition currently in place.^1^ The global burden of neurological and neurodevelopmental disorders is particularly high in low- and middle-income countries (LMICs). Neurological conditions are the second leading cause of death worldwide, responsible for approximately nine million deaths each year.^2^ These disorders can result from acute (short-term) or chronic (long-term) brain injuries, with long-term consequences having a profound impact on overall health.^3^,^4^

Brain function can be categorized into four primary domains: perception, action, cognition, and emotion. Therefore, a comprehensive approach to assessing brain health must consider all of these domains to form a holistic picture.^3^ Research has suggested that brain health encompasses five key functional domains: motor, cognition, emotions, hearing, and vision.^4^,^5^ In 2021, Boyle et al. introduced the concept of the “Cognitive Clock,” which suggests that cognitive age may provide a more accurate measure of brain health compared to chronological age.^6^ Cognitive clock is an excellent example of how chronological age is not the only parameter for measuring age. Our study took this observation and grew from there by including other parameters such as emotional wellbeing and motor function etc to gauge brain health.

Biomarkers have become valuable tools for diagnosis, prognosis, and treatment monitoring, providing insight into the underlying mechanisms of brain conditions.^7^ Ideally, biomarkers should be detectable in blood, offering a minimally invasive method of repeated assessment. A previously identified biomarker panel from a scoping review was used in this research for comparison.^8^ Despite extensive research, accurately measuring and monitoring brain health remains challenging. Effective tools must be sensitive, accurate, minimally invasive, multidimensional, and applicable across different demographics.^9^ Brain imaging, physiological tests, and biomarker assessments can offer valuable insights into brain health. However, the lack of consensus on the definition of brain health complicates the development of assessment tools and the integration of brain health into global healthcare strategies.^10^

With the global aging population, poor brain health has become a major public health issue, highlighting the need for early detection of brain damage. In LMICs such as Pakistan, cost-effective methods for assessing brain health are essential. Organizations like the Global Brain Health Institute are actively promoting brain health worldwide.^11^

Predictive modeling is crucial in diagnosing conditions, evaluating treatments, and assessing the long-term impact of diseases on individuals.^12^ However, no current model or assessment tool comprehensively addresses all aspects of brain health. The objective of this research was to develop a formula for measuring brain health by utilizing various brain faculties and a panel of plasma neurobiomarkers as predictors and criterion variables, respectively.

## METHODS

This was a predictive cross-sectional modelling study which followed the STROBE and the TRIPOD guidelines for reporting and extended from May 2022 to February 2023. The research work was carried out in various settings across Peshawar, Pakistan, including, Khyber Medical University (KMU) and Khyber Medical College (KMC).

### Ethical approval:

All procedures performed in studies involving human participants were in accordance with the ethical standards of the institutional and/or national research committee and with the 1964 Helsinki Declaration and its later amendments or comparable ethical standards. This research was approved by the ethical board of KMU vide no. KMU/IBMS/IRBE/meeting/2022/8072, dated: June 29, 2022.

The participants were selected from Peshawar, Pakistan through purposive sampling and were recruited through flyers, social media ads, personal contacts. A total of 75 participants were included for the study. Sample size was calculated using G*Power software. Effect size f² of 0.15 was taken, α error as 0.05, Power (1-β err prob) as 0.95 and number of predictors were taken as two. The No Objection Certificate (NOC) for data collection was obtained from Institutional Review and Ethical Board (IREB), KMC/KTH vide letter No: 228/DME/KMC.

A participant information sheet was provided to each participant and informed consent obtained. The study participants were healthy adults of ages 19-35 years no reported cardiac, renal, or hepatic conditions; free from psychiatric conditions; on no medications, alcohol, or drugs. Females on hormonal contraceptives were excluded. The exclusion criteria were; motor or cognitive deficits such as autism, moderate to severe hearing loss, grossly impaired vision and mechanical limitations such as fractures. In addition, individuals with diagnosed mental disabilities and history of chronic diseases were also excluded. Those individuals who smoked or had more than six cups of coffee or tea per day were also excluded.

### Data collection procedure:

The brain functions’ selection was based on the literature review and qualitative research done by Zafar et al.^13^ The score obtained from the rubric application led to identification of basic scales/tools for identification of brain faculties^8^ which were then used to devise the brain health formulae. Each subject was made to undergo tests for seven predictor modalities (vision, hearing, Cognition, Strength, Balance, Emotion, age). The formula was then devised using multiple linear regression equation:

***Equation-1:*** Linear Regression equation

Y_i_ = ß_o_ + ß_1_x_1_ + ß_2_x_2_ + …+ ß_k_x_k_ + ε

### Protocols for various tests:

Visual fields of each participant, was analyzed using Goldmann’s student perimeter. If vision was disrupted in one quadrant, loss was recorded as less than 25%, if in two quadrants then loss was between 25 and 50%, if in three quadrants than loss was between 50 and 70% and if all four quadrants were deranged, loss was considered greater than 75%. Normal field extents are superior and nasal 60°, inferior 70° and temporal 90°.

For hand grip strength test, the maximum voluntary contraction (MVC) was determined by asking the participant to apply maximum possible pressure to a dynamometer (T.K.K 5401) with his/her dominant hand in a seated or standing position with their elbow straight down. Three readings were taken and an average determined. The degree of hearing loss was quantitively assessed based on five categories using an audiometer. If there was 0 to 25 dB loss then participants were labelled normal.

Similarly, for 26 to 40 dB; mild loss, 41 to 60 dB; moderate loss, 61 to 80 dB; severe loss and 81 or above was taken as profound loss. TEIQue-SF is a 30 items questionnaire designed to measure global trait emotional intelligence (Trait EI). Based primarily on the correlations with total facet scores, it uses a 7-point Likert-style response option format, ranging from 1 = completely disagree to 7 = completely agree. A global trait EI score is calculated by summing up item scores and dividing by the total number of items. Similarly, MMSE is a 30-point questionnaire which is designed to measure cognitive function. TEIQue-SF and MMSE were administered to all the participants of the study. Balance was assessed using Romberg’s test but it was eliminated during the analysis due to lack of variance amongst the values for each subject.

### Neurobiomarkers’ measurement:

A panel of neuro-biomarkers (NfL, NSE, pTau) were measured for each participant of both phases, using 96 wells ELISA Kit (Cat. No. E-EL-H0741) of Elabscience. The technique used was Sandwich ELISA technique.

### Statistical Analysis:

The outcome predicted was brain health as a continuous variable. Standard descriptive statistics were applied for numerical variables (height, weight, age, blood pressure, heart rate, hand grip strength, NfL, NSE, pTau, MMSE and TEIQue SF scale) and frequencies were calculated for categorical variables (gender, visual fields and hearing). Multiple linear regression modelling was done after meeting the assumptions of no multicollinearity, homoscedasticity and normality; which were checked via variance inflation factor (VIF) values (less than 10) and scatter plot, and P-P Plots respectively. For doing the predictive modeling TRIPOD guidelines were followed to increase transparency and reproducibility.^14^ The predictive model performance was reported by considering R^2^ and Adjusted R^2^ and p value less than 0.05 was considered significant.^15^

### K-fold cross validation:

The data was allotted codes (1-75 randomly allotted to data points) in such a way that there were no obvious patterns of distribution of values of various variables like an ascending or a descending trend. The data was then transformed and divided into subsets. Training samples (1,2,3,4,5) were created having 25, 50, 26, 26, 26 data points each. Multiple linear regression was run on the whole sample using ENTER method and then on the each of the Training subsets. This was performed for all three neuro-biomarkers as predictor variables. The R^2^ and Adjusted R^2^ values were compared and if there was more than 2% difference the initial prediction model was deemed a poor predictor.^15^ With k = 5, each model is trained on 80% of the data and tested on the remaining 20%. This split typically achieves a low-variance estimate of performance without the high computational cost of leave-one-out cross-validation. Values of k between five and 10 are commonly recommended when the sample size (n) is in the low hundreds. (Hastie, Tibshirani & Friedman, ESL, 2 ed., 7.10; Kuhn & Johnson, Applied Predictive Modeling, 4.4). Kohavi et al. (1995) and later studies found that 5-fold and 10-fold CV give very similar error estimates on most real data sets; the difference is rarely statistically significant.^16^

### Inferential statistics:

The Paired sample T Test was applied to gauge the difference between the predicted and observed values of all three neuro-biomarkers in both data sets (healthy and diseased). The data management and analysis was done using SPSS version 26.0 for MacBook Pro.

### Patient involvement:

Participants were involved at several stages of the research, including the tests conduction, and filling the questionnaires. We intend to disseminate the main results to the general public via publishing the research findings.

## RESULTS

Out of the 75 healthy participants that agreed to participate in the study 50.7% were males and 49.3% were females. All the demographic and the main variables (age, NfL, NSE, pTau, MMSE, TEIQue SF and hand grip strength) were normally distributed. Majority of the participants had normal hearing 78.7% (n=59); 76% (n=57) had normal hearing in left ear and 96% (n=72) had normal hearing in right ear. Normal visual fields were present in 81.3% (n=61) individuals; 80% (n=60) had normal VF in left eye and 81.3% (n=61) in the right eye. The mean maximum hand grip strength in dominant hand was 30.43±8.49.

### Regression modeling:

The average score in the TEIQue-SF inventory was 144.72±19.47, and that in the MMSE was 29.97±0.16 (normal cognition is a score of 30). The mean NfL value was 0.026±0.011 ng/ml, NSE was 10.46±1.52 ng/ml, pTAU was 4.48±3.23 ng/ml (Table-I). Comparison of univariate with multivariate regression showed that only TEIQue-SF had significant association with NfL (Table-II). For NSE there were no significant associators (Table-III). For pTau age, handgrip strength and TEIQue-SF scores were significant in univariate analysis while in multivariate analysis only TEIQue-SF scores were significant (Table-IV).

**Table-I:** Demographics and main predictor variables for healthy sample.

Variable				Mean± SD/ frequency (%)		
BMI*				21.54±3.2		
Height				166.24±6.4		
Age				22.54±5.27		
Weight				59.8±5.27		
Gender	Male			38(50.7%)		
	Female			37(49.3%)		
Diastolic BP				73.11±10.22		
Systolic BP				114.51±13.77		
Heart rate				83.66±11.43		
*Hearing†*				*Hearing left ear*	*Hearing right ear*	*Overall hearing*
Normal				57(76%)	72(96%)	59(78.7%)
Mild				18(24%)	3(4%)	16(21.3%)
Moderate				0	0	0
Severe				0	0	0
Profound				0	0	0
*Visual fields^+^*				*Visual fields left eye*	*Visual fields right eye*	*Overall visual fields*
Normal				60(80%)	61(81.3%)	61(81.3%)
Less than 25% loss				15(20%)	14(18.7%)	14(18.7%)
25-50% loss				0	0	0
50-75% loss				0	0	0
Greater than 75% loss				0	0	0
Hand grip ^¤^ left hand				29.94±8.78		
Hand grip right hand				30.88±9.21		
Overall hand grip				30.43±8.49		
Dominant hand		Right		66(88%)		
		Left		9(12%)		
TEIQue-SF			144.72±19.47			
MMSE			29.97±0.16			
NfL (ng/ml)			0.026±0.011			
NSE (ng/ml)			10.46±1.52			
pTAU (ng/ml)			4.48±3.23			

Values are mean ± SD or number (%). *BMI: Body Mass Index, †Hearing was measured using audiometry, ^+^Visual, ^¤^ Hand grip strength, TEIQue-SF: Trait Emotional Intelligence Questionnaire Short Form, MMSE: Mini-Mental State Exam, NfL: Neurofilament Light chain, pTau: phosphorylated Tau proteins and, NSE: Neuron Specific Enolase (NfL, NSE and pTau were quantified using Enzyme-Linked Immunosorbent Assay (ELISA).

**Table-II:** Comparison of regression model 1 & 2 while keeping NfL as dependent variable.

Predictors	Univariate analysis (Model 1)		Multivariate analysis (Model 2)	
	Beta	p-value	Beta	p-value
Age	0.062	0.595	0.097	0.427
Hearing	-0.071	0.545	-0.061	0.604
Vision	0.026	0.825	-0.012	0.924
HG	-0.098	0.404	-0.212	0.098
TEIQue-SF	0.328	0.004	0.349	0.003

TEIQue-SF: Trait Emotional Intelligence Questionnaire Short Form, MMSE: Mini-Mental State Exam, HG: Hand grip, NfL: Neurofilament Light chain, pTau: phosphorylated Tau proteins and, NSE: Neuron Specific Enolase Dependent variable: NfL MMSE being constant was not included in the regression models (1&2) R^2^ for Model 2: 0.145, Adjusted R^2^ for Model 2: 0.083 Regression formula generated: NfL = 0.003 + 0.000 * Age - 0.002* Hearing + 0.000 * HG + 0.000 * TEIQue-SF. Excluded variable: Vision.

**Table-III:** Comparison of regression model 1 & 2 while keeping NSE as dependent variable.

Predictors	Univariate analysis (Model 1)		Multivariate analysis (Model 2)	
	Beta	p-value	Beta	p-value
Age	0.014	0.907	0.045	0.729
Hearing	0.049	0.676	0.043	0.73
Vision	-0.059	0.616	-0.094	0.47
HG	-0.094	0.425	-0.117	0.387
TEIQue-SF	-0.101	0.39	-0.076	0.534

TEIQue-SF: Trait Emotional Intelligence Questionnaire Short Form, MMSE: Mini-Mental State Exam, HG: Hand grip, NfL: Neurofilament Light chain, pTau: phosphorylated Tau proteins and, NSE: Neuron Specific Enolase Dependent variable: NSE MMSE being constant was not included in the regression models (1&2) R^2^ for Model 2: 0.027, Adjusted R^2^ for Model 2: -0.044 Regression formula generated: NSE = 11.918 - 0.013 * Age + 0.159 * Hearing - 0.365 * Vision - 0.21 * HG - 0.006 * TEIQue.

**Table-IV:** Comparison of regression model 1 & 2 while keeping pTau as dependent variable.

Predictors	Univariate analysis (Model 1)		Multivariate analysis (Model 2)	
	Beta	p-value	Beta	p-value
Age	0.252	0.029	0.122	0.299
Hearing	-0.114	0.329	-0.034	0.765
Vision	-0.103	0.381	0.028	0.237
HG	0.394	0.000	0.319	0.011
TEIQue-SF	0.258	0.025	0.182	0.104

TEIQue-SF: Trait Emotional Intelligence Questionnaire Short Form, MMSE: Mini-Mental State Exam, HG: Hand grip, NfL: Neurofilament Light chain, pTau: phosphorylated Tau proteins and, NSE: Neuron Specific Enolase Dependent variable: pTau MMSE being constant was not included in the regression models (1&2) R^2^ for Model 2: 0.204, Adjusted R^2^ for Model 2: 0.147 Regression formula generated: pTau = - 5.188 + 0.075 * Age - 0.263 * Hearing + 0.228 * Vision + 0.12 * HG + 0.030 * TEIQue.

### Cross checking the Regression Formulae against the observed values (Healthy sample):

During k-fold cross validation there was more than 2% difference between the R^2^ values of the main regression model and the training samples for all three neuro-biomarkers (Table-V). There was significant difference (p<0.001) between actual NfL, NSE and pTau values and those predicted by the formula devised via multiple linear regression.

**Table-V:** K-fold cross validation with NfL, NSE and pTau as dependent variables.

Neuro-biomarkers	Model	R^2^	Adjusted R^2^	p-value
NfL	Model 2	0.145	0.083	0.051
	Training sample 1	0.302	0.118	0.197
	Training sample 2	0.131	0.032	0.272
	Training sample 3	0.089	-0.138	0.849
	Training sample 4	0.248	0.059	0.297
	Training sample 5	0.093	-0.134	0.836
NSE	Model 2	0.027	-0.044	0.86
	Training sample 1	0.311	0.13	0.179
	Training sample 2	0.048	-0.06	0.816
	Training sample 3	0.047	-0.191	0.959
	Training sample 4	0.131	-0.087	0.700
	Training sample 5	0.054	-0.182	0.946
pTau	Model 2	0.204	0.147	0.007
	Training sample 1	0.387	.0225	0.076
	Training sample 2	0.226	0.138	0.04
	Training sample 3	0.195	-0.007	0.461
	Training sample 4	0.178	-0.027	0.519
	Training sample 5	0.217	0.021	0.387

## DISCUSSION

This study aimed to develop formulae for evaluating brain health by utilizing specific brain faculties as predictors and brain-specific neurobiomarkers as response variables. Multiple linear regression models were employed, using predictors such as age, hearing, vision, hand grip strength, TEIQue-SF, and MMSE scores.

The concept of “brain health” was first introduced in 1989, with early attempts to measure it based on intrinsic brain attributes.^17^ Erol Başar’s theory of whole-brain work draws parallels between brain waves and language generation, likening brain waves to the syntax of language. Brain functions, though inherently chaotic, can be simplified and quantified using EEG.^18^ Karl Friston suggested that the human brain operates as a hierarchical ‘prediction machine,’ refining its model of the world through cycles of perception and action aimed at reducing environmental uncertainty. Paul Badcock’s Hierarchically Mechanistic Mind (HMM) hypothesis further supports the idea of the brain functioning as an integrated hierarchy of local and global functions.^19^ This study supports the idea that when brain functions are normal, local functions such as vision, memory, and motor actions harmonize to achieve global brain function. When comparing predicted neurobiomarker values (NfL, NSE, pTau) with their actual values, we found significant discrepancies, indicating the limitations of the prediction models in estimating brain health. Our findings suggest that each predictor showed a significant association with the others, but their predictive power when used holistically diminishes. Thus, predicting overall brain health in healthy individuals using intrinsic brain functions yields poor results. This is consistent with Marcus E. Raichle’s perspective that intrinsic brain activity is a complex web of interrelated activities across multiple levels, including behavior, large-scale brain systems, and cellular properties. Raichle further proposed two approaches for measuring brain activity: a top-down approach (using fMRI, PET, and EEG) and a bottom-up approach (involving more invasive methods like studying histological slices of cortex in animal models).^20^

In our study, stable variables like vision and hearing in healthy adults were less effective predictors due to their limited variability. For instance, hearing thresholds in healthy adults tend to fall within a narrow range (0-25 dB), reducing its predictive utility.^21^ Complex systems like the brain involve multiple interdependent components, making predictability and regularity difficult to model when nonlinear interactions are at play.^22^ EEG-based complexity measurements, such as multiscale entropy (MSE), can capture these interactions in diseased states like schizophrenia, epilepsy, and autism spectrum disorder, as noted by Lau et al.^23^

Yang and Tsai characterized complexity in physiological functions as the result of intricate interactions between components at both microscopic and macroscopic levels.^24^ Our findings suggest that normal brain functions exhibit harmony and reduced diversity, which may account for the low predictive power of brain health models in healthy individuals. Paulus et al. demonstrated that patients with schizophrenia exhibited more irregular brain function patterns and higher predictability compared to healthy individuals, a finding that supports our results. Their dynamic entropy (h) values, calculated using machine learning algorithms, revealed a stronger linkage between neuro-behavioral measures and brain functions in schizophrenia patients than in normal subjects.^25^ Vaillancourt and Newell further postulated that the complexity of brain physiology may fluctuate with age and disease, based on the system’s intrinsic needs and dynamics.^26^ Thus, the harmony and reduced variability observed in healthy individuals may reflect the system’s requirement to carry out necessary functions efficiently.

### Strength of this study:

The strength of this study include its Multidomain design, holistic model testing, healthy baseline sample and transparent negative results. This is the first study to test whether easily obtained functional metrics (vision, hearing, grip strength, emotional intelligence, and cognition) can stand in for laboratory-based brain-specific biomarkers (NfL, NSE, pTau) in neurologically intact adults. By showing that even a multivariate model explains only a small fraction of biomarker variance, our work sets an empirical upper limit on the accuracy of function-only screening tools. It therefore challenges the implicit assumption—common in both geriatric screening and digital-health discourse—that “simple bedside tests” can reliably index molecular brain integrity.

### Limitations:

Some of the limitations include omission of education level, sleep, and physical activity as factors that may influence TEIQue or MMSE. Predictive accuracy could be improved by increasing the sample size in future researches. Or by cross checking the parameters like cognition and emotional wellbeing with more objective assessment methods like CANTAB cognition or NIH toolbox.

## CONCLUSION

In this small sample of healthy young adults, regression models combining functional tests and plasma biomarkers failed to yield accurate estimates based on individual biomarker concentrations. Larger, more diverse cohorts and nonlinear modelling approaches are needed before discarding functional metrics as potential proxies of brain health. The reason being that healthy brain’s functions are nearly constant in terms of computation and they augment each other leading to global brain function.By constant we mean lack of variability in some of the brains functions’ values among the subjects, like vision and balance.

### Author contributions:

**UZ and SHH:** Drafted, participated in manuscript preparation, the design of the study, critical review of the manuscript on several stages. **MOM:** Performed the statistical analysis. **UZ:** participated in data collection. Is responsible for accuracy of the data and integrity of the study.
